# Changing speed along the mental line: A SNARC-like effect for visual acceleration

**DOI:** 10.3758/s13423-026-03016-0

**Published:** 2026-10-05

**Authors:** Mario Dalmaso, Stefano Dalla Bona, Michele Vicovaro

**Affiliations:** 1 Department of Developmental and Social Psychology, University of Padova, Via Venezia 8, 35131 Padova, Italy; 2 Department of General Psychology, University of Padova, Via Venezia 8, 35131 Padova, Italy

**Keywords:** SNARC-like effect, Visual acceleration, Spatial representation

## Abstract

Humans tend to map magnitudes onto space, with smaller values associated with the left and larger values with the right. Recent evidence suggests that such spatial mappings extend beyond numbers to include visual speed. Whether a second-order motion property, such as visual acceleration, is also spatially represented remains unclear. We addressed this question in two experiments using a centrally presented random dot kinematogram (RDK). Participants indicated, via lateralised key presses, whether the stimulus decelerated or accelerated relative to an initial constant-speed reference. In Experiment 1, all dots moved coherently in a single direction randomly selected on each trial. In Experiment 2, dot directions were randomised at the element level, creating an isotropic motion field. A distance effect (i.e., faster responses for larger absolute acceleration values) emerged in both experiments, indicating that acceleration was processed as a continuous magnitude. Crucially, a left-to-right spatial mapping emerged only in Experiment 2. These findings provide evidence that visual acceleration can be spatially represented, but that this mapping depends on the structure of the motion signal.

## Introduction

The human mind systematically organises both numerical and non-numerical magnitudes along spatial continua. This phenomenon is famously captured by the Spatial-Numerical Association of Response Codes (SNARC) effect, whereby small numbers are typically associated with the left side of space and large numbers with the right side (Dehaene et al., 1993). Importantly, such spatial-numerical associations are not fixed but can be shaped by cultural conventions, particularly reading and writing direction. For instance, it has been shown that among Arabic or Farsi speakers (two languages written from right to left), space–number associations can follow a right-to-left orientation (Dehaene et al., 1993; Shaki et al., 2009; but see, e.g., Bulut et al., 2025; Hochman et al., 2025). Beyond numbers, similar SNARC-like effects have been consistently observed for a broad range of non-numerical magnitudes, including time (e.g., Ishihara et al., 2008; Mariconda et al., 2022), physical size (e.g., Ren et al., 2011; Sellaro et al., 2015), weight (e.g., Vicovaro & Dalmaso, 2021), and even social categories such as face age (e.g., Dalmaso et al., 2023a, 2026b). These findings (see also Macnamara et al., 2018, for a review and meta-analysis) suggest the existence of a generalised neuro-cognitive system for magnitude processing and representation, as proposed by ‘A Theory of Magnitude’ (ATOM; Walsh, 2003).

Recent research has extended this framework to the domain of visual speed. In a series of experiments, Vicovaro et al. (2025) asked Western participants to classify the speed of a centrally presented random dot kinematogram (RDK), relative to a reference, by pressing either a left or right button. The main finding was that slower speeds were associated with the left side of space and faster speeds with the right. Crucially, this mapping proved robust across different motion conditions, emerging not only when stimuli moved consistently from left to right, but also when motion was randomised across trials or even when dots moved from right to left. These results indicate that speed, a magnitude inherently tied to both space and time, is nonetheless represented along a left-to-right continuum. In addition, a distance effect emerged consistently, with faster responses for higher velocities, supporting the presence of a graded representation of speed. Recently, a right-to-left representation of speed (and a distance effect) has been documented in a sample of Iranian participants (Dalmaso et al., 2026a), further underscoring the role of cultural habits in shaping the spatial representation of magnitudes.

Speed, however, is not the only kinematic variable relevant to human perception and action. A fundamental aspect of motion is its rate of change, namely acceleration. Critically, unlike speed, acceleration is a second-order property of motion, reflecting changes in velocity over time rather than an instantaneous state. Moreover, unlike speed and most other physical dimensions, acceleration is a signed quantity, with negative values indicating a decrease in speed (i.e., deceleration), while positive values indicate an increase in speed. In physical terms, acceleration reflects forces acting upon objects and allows predictions about future positions. In psychological terms, it provides crucial cues for a variety of perceptual and cognitive processes, such as anticipating events, inferring intentions, and adapting motor responses. These processes are essential for many everyday activities, from catching a ball in sport to driving a car. Notably, at the perceptual level, acceleration appears to be less strongly represented than speed (e.g., Brouwer et al., 2002; Kreyenmeier et al., 2022; Mueller & Timney, 2016; Watamaniuk & Heinen, 2003), possibly reflecting the absence of neural populations specifically tuned to visual acceleration (e.g., Price et al., 2005). Interestingly, sensitivity to acceleration appears to vary as a function of the complexity of the motion pattern, being higher for more complex patterns compared to simpler, unidirectional ones (Mueller & Timney, 2016). This raises the question of whether such a second-order property can be spatially represented at all, and under which conditions.

In the present work, we investigated whether visual acceleration is spatially mapped in a SNARC-like fashion, with the aim of understanding whether and how second-order motion properties are encoded within spatial frameworks. If negative and positive acceleration values are mapped onto a common spatial continuum, and assuming that negative acceleration is represented as ‘less speed/energy’ and positive acceleration as ‘more speed/energy’, one would expect negative values to be associated with the left side of space and positive values with the right (at least in Western populations), as well as a distance effect. Alternatively, negative and positive accelerations may be perceived as qualitatively distinct dimensions that do not share a common spatial representation, which would predict no SNARC-like effect. To test these hypotheses, we conducted a first experiment based on Experiment 2 of Vicovaro et al. (2025). Specifically, all dots within the RDK moved coherently in the same direction, which was randomly determined on each trial across the full 360° range. This approach ensured that no systematic directional cues were available, allowing any spatial association to be more reliably attributed to acceleration processing rather than to consistent motion signals along a specific axis.

## Experiment 1

### Methods

#### Participants

Sample size was determined a priori following guidelines for linear mixed models (Brysbaert & Stevens, 2018), which recommend at least 1,600 observations per experimental condition, resulting in a minimum of approximately 27 participants. Data collection terminated at the end of a booking session, with a final sample of 34 participants (mean age = 22.1 years, *SD* = 1.31; eight males; all self-reported right-handed), all students at the University of Padova. As this is the first study investigating a SNARC-like effect for visual acceleration, a more precise design analysis was not feasible. However, the present sample size is comparable to that used in previous studies on the SNARC-like effect for visual speed (Dalmaso et al., 2026a; Vicovaro et al., 2025), where it proved sufficient to adequately control both Type I and Type II errors. Manual preference was assessed using the short form of the Edinburgh Handedness Inventory (EHI; Veale, 2014), with a mean score of 81.25 (*SD* = 20.47; 30 right-handed, four mixed-handed). Given the limited variability in handedness within the sample, the possible impact of this variable was not further explored.

#### Apparatus, stimuli and procedure

The task, programmed in PsychoPy3 (Peirce et al., 2022), was presented on a 52 cm (width) × 29 cm (height) LCD monitor (1,920 × 1,080 pixels; 60 Hz). Participants sat about 70 cm from the screen and responded using a centrally placed standard keyboard. Screen background was set to grey.

**Fig. 1:**
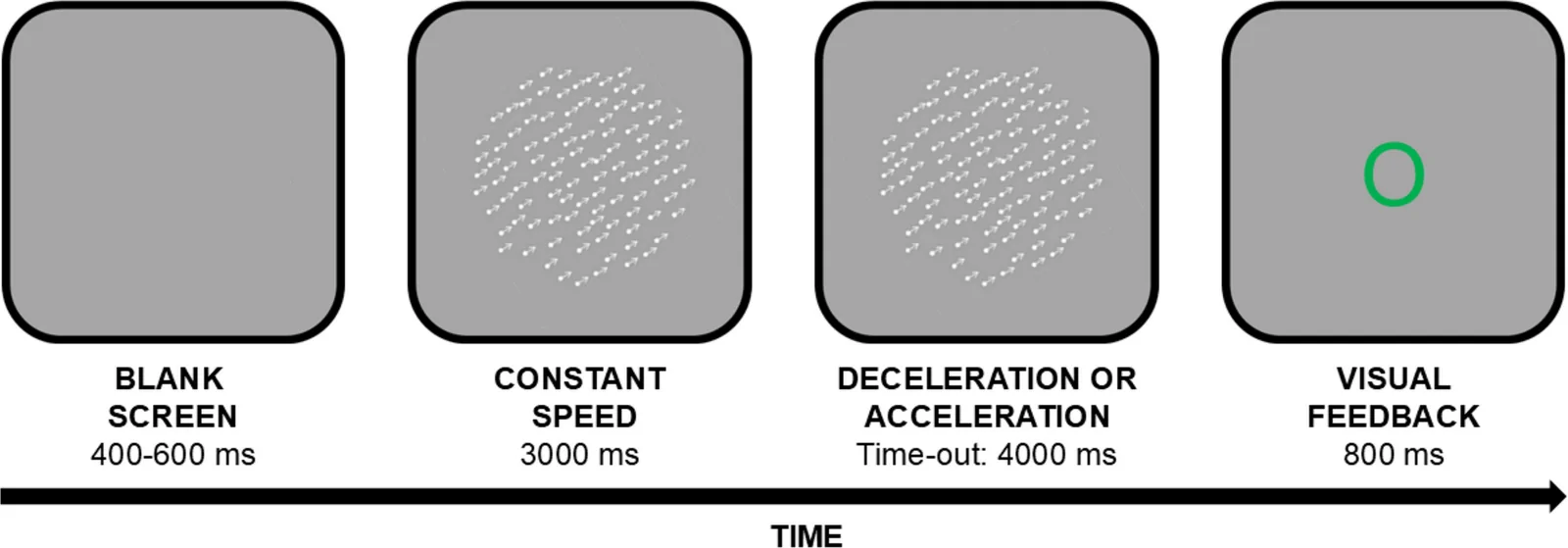
Illustration of a trial and the stimuli (not to scale) used in the experiments. The arrows shown in the random dot kinematogram (RDK) indicate a possible motion direction (in this example, all dots move in the same direction, as in Experiment 1) and are included for illustrative purposes only. The visual feedback ‘O’ indicates a correct response

The experimental stimulus was an RDK (see Fig. 1) generated with the ‘dots’ tool of the PsychoPy3 Builder. Each RDK consisted of 100 white dots moving within a circular area (10 cm diameter). On each trial, the direction of the dots varied randomly from 0° to 360° (in each trial, all dots always moved in the same randomly chosen direction). Dots had a size of 5 pixels and a lifespan of 60 frames (1 s): after this time, or if they moved outside the circular area, they were replaced at a random location within the area. This procedure ensured that the number of dots displayed remained constant throughout the stimulus presentation.

Each trial started with a blank screen presented for a random duration between 400 and 600 ms. An RDK was then displayed with a constant motion speed randomly selected between 0.07 and 0.13 deg/frame. After 3,000 ms, the speed of the RDK progressively changed over time, for a maximum duration of 4,000 ms, resulting in a total trial duration of 7,000 ms. From the onset of this change, the elapsed time was used to update the dot speed according to the assigned acceleration (negative for deceleration, positive for acceleration), resulting in a continuous increase or decrease in speed across frames. To avoid non-physical values, a minimum speed threshold of 0.01 deg/frame was enforced. Acceleration values were randomly drawn from a set ranging between ± 0.0105 and ± 0.015 deg/frame^2^ in increments of 0.0005 (i.e., 10 negative and 10 positive values). Participants were instructed to indicate whether the RDK was decelerating or accelerating by pressing the ‘S’ or ‘K’ keys. In one block, ‘S’ corresponded to deceleration and ‘K’ to acceleration, whereas in the other block the mapping was reversed (counterbalanced across participants). If no response was provided within 7,000 ms, visual feedback appeared centrally saying ‘TOO SLOW’; otherwise, a green ‘O’ appeared for correct responses and a red ‘X’ for incorrect responses. The two experimental blocks (120 trials each) were each preceded by a practice block (10 trials). Within each experimental block, each experimental stimulus was presented an equal number of times and in a random order. Finally, participants completed a computerised version of the EHI (Veale, 2014).

**Fig. 2:**
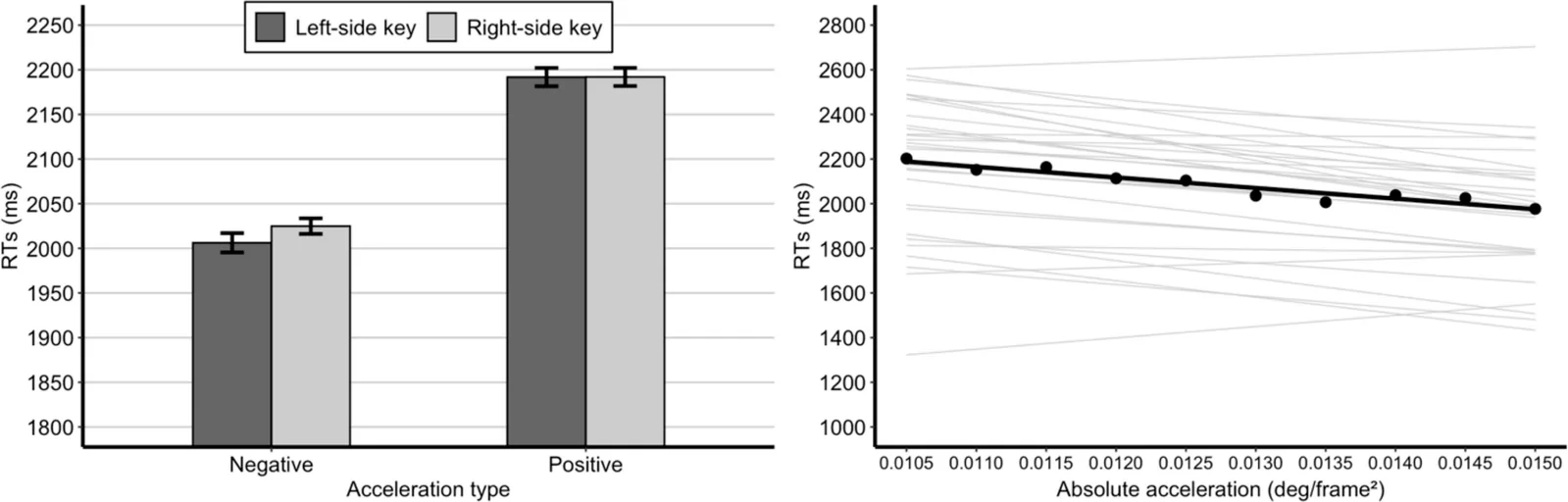
Results of Experiment 1. The left panel depicts model-based estimates of mean reaction times (RTs), shown as a function of acceleration type and response side, with error bars representing the estimated *SEM*. The right panel depicts the distance effect, including the estimated fixed effect (thick line), random effects (thin lines), and observed mean RTs (dots)

### Results

Data were analysed with R (https://cran.r-project.org/). Trials without a response within the 7,000-ms timeout limit (2.81%) or with responses provided before the 3,000-ms acceleration/deceleration onset (9.26%) were discarded and not further analysed. Trials with incorrect responses (9.61%) were excluded from the reaction time (RT) analyses and analysed separately. Four participants with less than 50% correct responses were excluded. Correct trials with latencies shorter than 200 ms or exceeding three standard deviations from the mean of each participant were treated as outliers and removed (0.84%). As for RTs, we fit a series of generalised linear mixed models (GLMMs) with a Gamma distribution and identity link to analyse correct RTs (Bates et al., 2015), as RTs are positively skewed (Lo & Andrews, 2015; see also, e.g., Dalmaso et al., 2026a; Vicovaro et al., 2025). In addition, acceleration type was treated as a categorical variable, consistent with the assumption that, in explicit magnitude classification tasks, the relationship between target magnitude and RTs follows a step function rather than a continuous one (Gevers et al., 2006; see also, e.g., Dalmaso et al., 2026a; Vicovaro et al., 2025). Models with increasing random-effects complexity were compared using the Akaike Information Criterion (AIC). The best-fitting model included acceleration type (negative vs. positive), response side, and their interaction as fixed effects. Random effects comprised intercepts for participants and acceleration values, random slopes for acceleration type, response side, and their interaction by participant, and random slopes for response side by acceleration values. The model was then evaluated using a Type III ANOVA and estimated marginal means. A significant main effect of acceleration type emerged, *χ*^*2*^(1) = 161.02, *p *< .001, with faster responses for negative (*M *= 2,016 ms, *SE* = 9.03) than positive acceleration (*M *= 2,192 ms, *SE *= 7.86). Neither the main effect of response side, *χ*^*2*^(1) = 0.82, *p *= .365, nor the interaction between acceleration type and response side, *χ*^*2*^(1) = 0.61, *p *= .435, was significant.

To test the presence of a distance effect (i.e., faster RTs for larger absolute acceleration values), two GLMMs (Gamma, identity link) differing in their random-effects structure were compared using AIC, with the best-fitting model including absolute acceleration as a continuous predictor, and a maximal random-effects structure with by-participant random intercepts and slopes for absolute acceleration. A significant negative effect of absolute acceleration emerged, *b *= − 47,006.13, *SE *= 5.16, *z *= − 9,107.9, *p *< .001, indicating a distance effect (the large estimate and z value reflect the small scale of the acceleration predictor).

Finally, wrong responses were analysed using binomial GLMMs with a logit link, following the same model-building strategy as for RTs. The best-fitting model was identical to that selected for RTs. No significant effects emerged (*p*s > .13).

### Discussion

The results clearly showed a distance effect, indicating that absolute visual acceleration was represented as a continuous mental magnitude. However, no SNARC-like effect emerged, suggesting that, although this dimension was encoded, it was not mapped onto the left–right spatial continuum. This finding is compatible with two hypotheses. First, negative and positive acceleration values may be perceived as two qualitatively distinct dimensions and, therefore, may not be spatially mapped onto a common continuum. Second, acceleration values may not have been sufficiently differentiated at the perceptual level to elicit a clear spatial representation of visual acceleration.

As mentioned in the *Introduction*, there is evidence suggesting that the perception of acceleration is less precise than that of speed (e.g., Brouwer et al., 2002; Kreyenmeier et al., 2022; Mueller & Timney, 2016; Watamaniuk & Heinen, 2003). Of particular interest for the current study is the work by Mueller and Timney (2016), who investigated visual acceleration perception using random dots, and compared performance across different motion patterns. In their study, participants were required to indicate which of two stimuli presented in sequence was changing speed (i.e., decelerating or accelerating). The results indicated higher sensitivity to acceleration when the motion patterns were relatively more complex, such as radial optic flow (characterised by dots moving in expanding or contracting patterns), as compared to simpler translational motion (characterised by dots moving leftwards or rightwards). According to Mueller and Timney (2016), this effect may reflect the involvement of higher-level motion-processing mechanisms, potentially linked to areas such as the Medial Superior Temporal area, which would be more strongly engaged by complex motion patterns, and this, in turn, may facilitate the extraction of a second-order motion property such as acceleration.

Following this rationale, we reasoned that, to reveal a SNARC-like effect for visual acceleration, participants should be immersed in a perceptual context favouring the processing of this kinematic dimension and, in turn, increasing the likelihood of its spatial mapping. Hence, we developed a second experiment in which, as opposed to Experiment 1, dot directions were randomised at the element level, thus creating an isotropic rather than a coherent motion field. In so doing we expected, in addition to replicating the distance effect observed in Experiment 1, to reveal a SNARC-like effect for visual acceleration.

## Experiment 2

### Methods

#### Participants

Thirty-four new participants (mean age = 21.7 years, *SD *= 0.88; ten males; three self-reported left-handed) were tested, all students at the University of Padova. The mean EHI score was 66.91 (*SD *= 47.16), with 28 participants classified as right-handed, four as mixed-handed, and two as left-handed. As in Experiment 1, given the limited variability in handedness within the sample, the possible impact of this variable was not further explored.

#### Apparatus, stimuli and procedure

Everything was identical to Experiment 1, except that each dot moved independently along a randomly assigned direction. Unlike Mueller and Timney (2016), we avoided expansion and contraction patterns, as these would have introduced a sagittal component (e.g., back-to-front) that could interfere with the horizontal mapping of interest (see also, e.g., Aleotti et al., 2020; Dalmaso et al., 2023a).

**Fig. 3:**
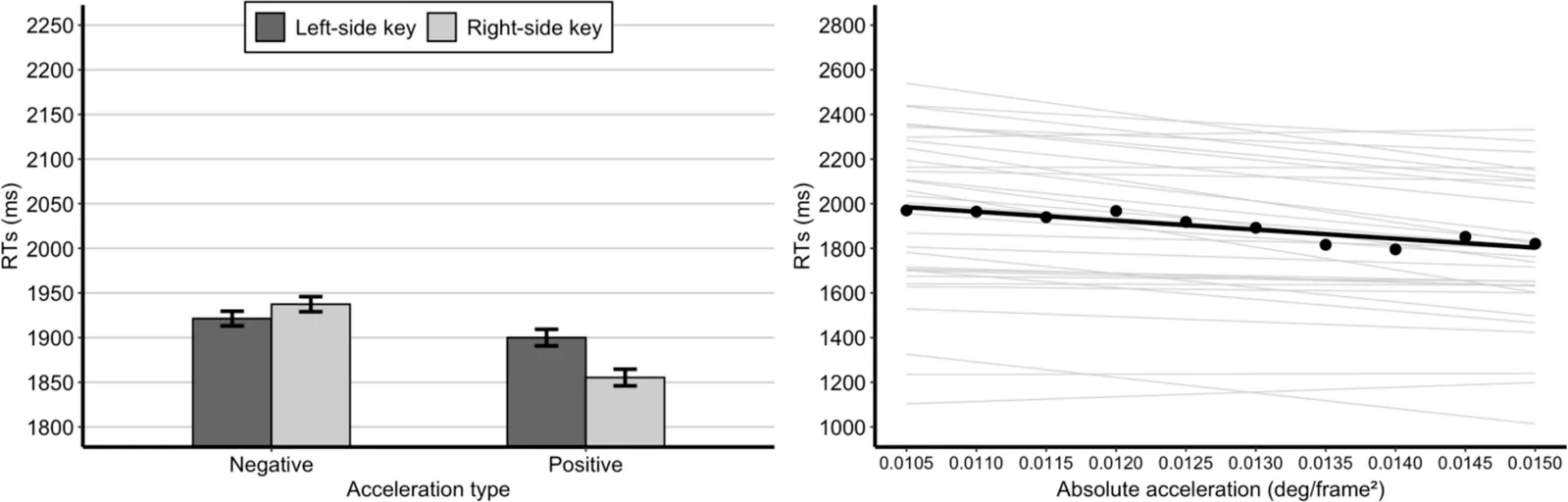
Results of Experiment 2. The left panel depicts model-based estimates of mean reaction times (RTs), shown as a function of acceleration type and response side, with error bars representing the estimated *SEM*. The right panel depicts the distance effect, including the estimated fixed effect (thick line), random effects (thin lines), and observed mean RTs (dots)

### Results

Data were analysed as in Experiment 1.

Trials with missing (1.10%) or anticipatory responses (6.99%) were discarded and not further analysed. Trials with incorrect responses (10.12%) were excluded from the RT analyses and analysed separately. Two participants with less than 50% correct responses were excluded. Correct trials with latencies shorter than 200 ms or exceeding three standard deviations from the mean were treated as outliers and removed (1.25%).

As for RTs, the best-fitting model included acceleration type, response side, and their interaction as fixed effects, random intercepts for participants and acceleration values, random slopes for acceleration type and response side by participant, their interaction by participant, and random slopes for response side by acceleration values. A significant main effect of acceleration type emerged, *χ*^*2*^(1) = 20.68, *p *< .001, with faster responses for positive (*M *= 1,878 ms, *SE *= 6.24) than negative acceleration (*M *= 1,929 ms, *SE *= 7.10). The main effect of response side was not significant, *χ*^*2*^(1) = 2.86, *p *= .091. Crucially, the interaction between acceleration type and response side was significant, *χ*^*2*^(1) = 11.89, *p *< .001. Pairwise comparisons revealed that, for positive acceleration, responses were faster with the right-side key (*M *= 1,855 ms, *SE *= 8.71) than with the left-side key (*M *= 1,900 ms, *SE *= 8.45), *z *= 3.78, *p *< .001, whereas no difference between the left-side key (*M *= 1,921 ms, *SE *= 8.00) and the right-side key (*M *= 1,938 ms, *SE *= 10.80) emerged for negative acceleration, *z *= − 1.29, *p *= .197, although the means were in the expected direction.

The distance effect was confirmed by the best-fitting model including absolute acceleration as a continuous predictor, random intercepts for participants, and by-participant random slopes for absolute acceleration. A significant negative effect of absolute acceleration emerged, *b *= − 39,533.86, *SE *= 3.305, *z *= − 11,960.7, *p *< .001 (the large estimate and z value reflect the small scale of the acceleration predictor).

Finally, as for wrong responses, the best-fitting model was identical to that selected for RTs. A significant main effect of acceleration type emerged, *χ*^*2*^(1) = 41.09, *p* <.001, with fewer errors for positive (*M *= 5.82%, *SE *= 0.82%) than negative acceleration (*M *= 11.40%, *SE *= 1.45%). No other significant results emerged (*p*s > .527).

### Discussion

The results confirmed the presence of a distance effect, consistent with Experiment 1. Crucially, they also revealed a left-to-right spatial representation of visual acceleration, with negative acceleration values represented to the left, and positive acceleration values represented to the right. This confirms that the nature of motion patterns may represent a key variable in modulating the perception of acceleration (see also Mueller & Timney, 2016) and, as indicated by the present experiment, also its spatial mapping.

## General discussion

We tested whether visual acceleration can be mapped onto space. In two experiments, participants classified whether a centrally presented RDK stimulus decelerated or accelerated by pressing a left or right response key. In Experiment 1, all dots moved coherently in a single direction randomly selected from 0° to 360°, whereas in Experiment 2 dot direction was randomised at the element level, creating an isotropic motion field.

In both experiments, a distance effect emerged (Moyer & Landauer, 1967), with faster responses for larger absolute differences between the target acceleration and the reference. This finding is consistent with previous results on visual speed (Vicovaro et al., 2025) and, more generally, with a large body of work showing similar effects across domains such as number (e.g., Murgia et al., 2026) and time (e.g., Dalmaso et al., 2023b). The presence of a distance effect indicates that absolute acceleration was processed as a continuous magnitude. In addition, in both experiments a significant main effect of acceleration type emerged, although its direction differed across conditions (i.e., faster responses for negative acceleration in Experiment 1 and for positive acceleration in Experiment 2), suggesting an asymmetrical processing of the target dimension. This pattern was unexpected. Considering that Mueller and Timney (2016) found no difference between the detection of negative and positive acceleration, this asymmetry would seem to depend on the specific motion condition. A speculative explanation could be that positive accelerations would be difficult to detect against the strong global motion signal in Experiment 1 but would emerge as added motion energy in the isotropic field of Experiment 2, thus becoming easier to detect. Future studies could further investigate this asymmetry, for instance by adopting psychophysical approaches based on perceptual thresholds to directly assess sensitivity to deceleration and acceleration under different motion conditions.

As for the presence of a spatial representation of acceleration – which was of primary interest – this was absent in Experiment 1 but emerged in Experiment 2, as indicated by the significant interaction between acceleration type and response side, which is typically considered the hallmark of SNARC and SNARC-like effects. Follow-up comparisons showed that only the effect for positive acceleration reached the conventional level of significance, whereas the effect for negative acceleration, although in the expected direction, did not. This asymmetry is not uncommon in the SNARC-like literature and may reflect the generally less robust spatial organisation of non-numerical magnitudes compared to numerical ones, as reported in several previous studies (e.g., Chang & Cho, 2015; Giuliani et al., 2024; Mariconda et al., 2026; Ren et al., 2011). In particular, Mariconda et al. (2026) reported a similar asymmetry for musical tempo and explained it through the polarity correspondence principle (Proctor & Cho, 2006), according to which ‘fast’ and ‘right’ would be coded as positive polarities and ‘slow’ and ‘left’ as negative polarities, with positive polarities being processed more efficiently. The same reasoning could apply here, although future studies are required to determine whether, and how, polarity correspondence may contribute to this spatial mapping. Nevertheless, a possible role of handedness cannot be excluded. Indeed, although the main effect of response side was not significant, responses tended to be faster with the right-side key, which may have amplified the effect for positive acceleration and reduced the effect for negative acceleration. In future studies, it would also be interesting to investigate whether and how negative and positive acceleration values are spatially represented when presented in separate blocks. For instance, participants could be required to classify negative (or positive) acceleration values as lower or higher than a negative (or positive) reference acceleration, rather than classifying the stimuli as decelerating or accelerating, as in the present studies. However, an important finding of Experiment 2 is that negative and positive acceleration values can be mapped onto a common spatial continuum, despite being qualitatively different at a perceptual level.

As for the discrepancy in the SNARC-like effect observed between our two experiments, it appears consistent with Mueller and Timney (2016), who showed that more complex motion patterns enhance the detection of acceleration compared to simpler ones. One possible explanation is that the extraction of visual acceleration relies on higher-level motion-processing mechanisms, which are more strongly engaged by complex motion patterns. In line with this view, isotropic motion in Experiment 2 may have provided a perceptual context that better supported the processing of acceleration as a second-order property, thereby allowing it to be represented as a spatially organised magnitude. In contrast, the more uniform motion signal in Experiment 1 may have been less effective in engaging these mechanisms, limiting the emergence of a spatial mapping.^1^ This interpretation, which is also supported by evidence of faster RTs in Experiment 2 than in Experiment 1 (see the comparison between Figs. 2 and 3), would align with meta-analytic evidence (Macnamara et al., 2018) showing that SNARC-like effects become stronger when a dimension is processed in a more relevant way (i.e., explicitly rather than implicitly), so that a more robust processing of acceleration could favour its spatial mapping. From a broader perspective, these findings support the idea that visual acceleration is more difficult to process than first-order motion properties (e.g., Brouwer et al., 2002; Kreyenmeier et al., 2022; Watamaniuk & Heinen, 2003), and that specific perceptual conditions may be required for its spatial organisation. In sum, the present findings show that visual acceleration can be mapped onto space. This supports the idea that even a signed spatio-temporal dimension can give rise to a SNARC-like effect.

## Footnotes

1 An alternative explanation is that, in Experiment 1, the coherent global motion signal may have been more salient than the acceleration signal, thereby reducing the likelihood that the latter was mapped onto space (see also Guida & Campitelli, 2019).

## Declarations

### Open practices statement

All experiment codes, data, and materials are available on the Open Science Framework: https://doi.org/10.17605/OSF.IO/JV6U9. The study was not pre-registered.
